# Hot-Air Flow Rolling Dry-Blanching Pretreatment Improves the Drying Quality of *Acanthopanax sessiliflorus* by Increasing the Drying Rate and Inactivating Enzymes

**DOI:** 10.3390/foods11203186

**Published:** 2022-10-13

**Authors:** Nan-nan An, Shi-yu Zhao, Dong Li, Yong Wang, Li-jun Wang

**Affiliations:** 1 College of Engineering, Beijing Advanced Innovation Center for Food Nutrition and Human Health, National Energy R & D Center for Non-Food Biomass, China Agricultural University, Beijing 100083, China; 2 College of Food Science and Nutritional Engineering, Beijing Key Laboratory of Functional Food from Plant Resources, China Agricultural University, Beijing 100083, China; 3 School of Chemical Engineering, The University of New South Wales, Sydney, NSW 2052, Australia

**Keywords:** blanching, *Acanthopanax sessiliflorus*, drying, polyphenol oxidase, peroxidase, microstructure

## Abstract

The processing of *Acanthopanax sessiliflorus* has attracted interest due to its health benefits. In this work, an emerging blanching technology, called hot-air flow rolling dry-blanching (HMRDB), was employed to treat *A. sessiliflorus* before drying. The effects of varied blanching times (2–8 min) on enzyme inactivation, drying characteristics, bioactive compound retention, and microstructure were examined. The results demonstrated that blanching for 8 min rendered polyphenol oxidase and peroxidase nearly inactive. The blanching process reduced the drying time of samples by up to 57.89% compared to an unblanched sample. The Logarithmic model showed good fitting performance for the drying curves. The total phenolic and flavonoid content of the dried product increased as blanching time increased. The total anthocyanin content of the samples blanched for 6 min was 3.9 times higher than that of the unblanched samples, and 8 min of blanching produced the greatest DPPH• and ABTS• scavenging capabilities. The retention of active compounds in a dried product is a result of the inactivation of enzymes and a reduced drying period. Changes in the porous structure of the blanched samples would be responsible for the accelerated drying rate, according to microstructural analysis. These results indicate that HMRDB enhances the drying process and improves drying quality when applied to *A. sessiliflorus* before drying.

## 1. Introduction

*Acanthopanax sessiliflorus* (Rupr. et Maxim.) Seem. is a plant of the Araliaceae family, which is widely cultivated in Korea, Japan, China, and the far-eastern region of Russia [1]. The rhizomes, leaves, and fruits of *A. sessiliflorus* are used for medicinal and culinary purposes, and are also traditionally used to prevent diabetes, hypertension, cancer, rheumatism, and allergies [2,3]. Many of the bioactive substances in *A. sessiliflorus* may contribute to bioactive functionality, including anthocyanins, phenolic acids, flavonoids, essential oils, and triterpenoids [4]. The moisture content of harvested *A. sessiliflorus* is high and susceptible to spoilage due to microbial infestation or mechanical damage. Therefore, drying is an important processing step for high moisture *A. sessiliflorus* to extend shelf life and reduce transportation and storage costs [5]. *A. sessiliflorus* fruits are usually dried by sun drying, hot air, and freeze drying, with dried products traditionally used as ingredients in wine or tea [6]. The skin of the fruits can prevent the migration of water during the drying process, resulting in a lower drying rate [7].

Blanching is a typical step prior to drying of food that deactivates enzymes, increases the drying rate, and reduces energy consumption [8,9]. Conventional blanching methods involve the use of hot water or vapor. Particularly, hot water blanching is the most popular and widely used method in the food industry due to low cost and simple operation [10]. However, hot water blanching may cause a loss in soluble nutrients (vitamins and bioactive compounds) via dissolution into the water and may generate wastewater that pollutes the environment [11]. Steam blanching can effectively reduce the loss of nutrients but its efficiency is low due to low heat transfer efficiency and uneven heating [11]. There is a need to develop an efficient blanching technology that increases quality and improves the processing efficiency of *A. sessiliflorus*.

**Figure 1:**
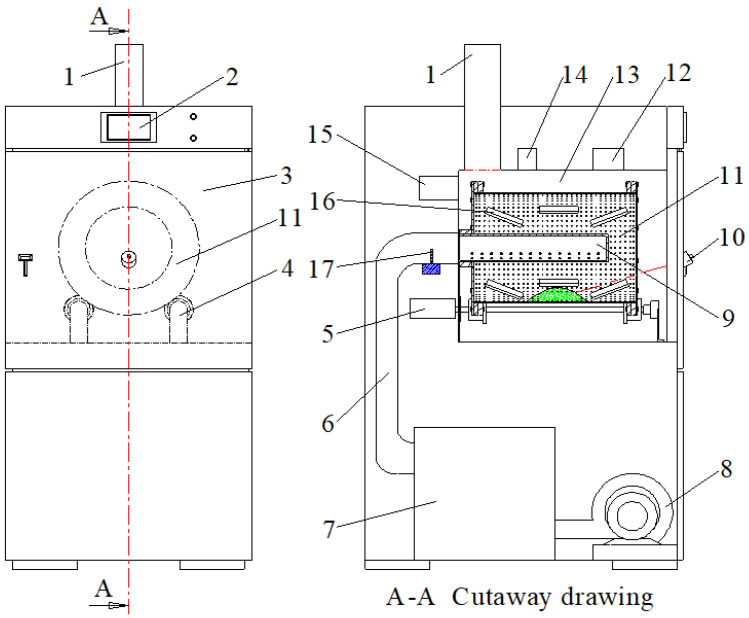
ORW2S-3000R microwave hot-air flow rolling dryer. 1—exhaust pipe; 2—touch screen; 3—resonant cavity door; 4—support frame; 5—drive motor; 6—hot-air pipe; 7—heat booster; 8—blower; 9—air dispersing tube; 10—infrared temperature sensor; 11—rolling bed; 12—microwave generator (A); 13—resonant cavity; 14—microwave generator (B); 15—control system; 16—agitating blades; 17—hot-air temperature sensor.

A microwave is an electromagnetic wave with a wavelength between 1 mm and 1 m, which is widely used in the food industry for drying, blanching, extracting, thawing, and sterilizing [12]. Microwave blanching is an efficient technology that has been suggested as an alternative to traditional hot blanching methods [12,13]. Previous studies have been reported on the application of microwave blanching in turnip slices [14], potato [13], and fruit peelings [15]. Compared to traditional blanching technology, the advantages of microwave blanching include high heat transfer efficiency, easy control, and less leaching of water-soluble nutrients. Dev et al. [16] found that microwave blanching reduced the drying time of raisins by 20% while maintaining good quality. Jiang et al. [17] compared hot water blanching and microwave blanching of *Agaricus bisporus* slices and found that the drying time was reduced by 25% and 41%, respectively. Increased total phenolic content and antioxidant properties of some fruit peels after microwave blanching were observed [15]. In order to improve the uniformity and heat transfer efficiency of microwave heating, our lab designed innovative hot-air microwave flow rolling dry-blanching (HMRDB) equipment, as shown in Figure 1. It was mainly composed of a microwave source, hot-air generating device, control circuit, and rolling bed. The material is heated simultaneously by the microwave and hot air, and the power of the microwave can be continuously adjusted in the range of 100 to 2000 W. Su et al. [18] reported that HMRDB improved the quality properties of *Pleurotus eryngii* and shortened the drying time.

Polyphenol oxidase (PPO) and peroxidase (POD) are widely found in plant tissues, and they may cause color changes, flavor loss, and nutrient degradation rapidly after harvest [8]. The inactivation efficiency of PPO and POD is commonly used to assess the effectiveness of the thermal blanching process. Deng et al. [9] found that PPO and POD activity in apricots was closely related to phytochemical content and antioxidant properties. Siguemoto et al. [19] and Okonkwo et al. [13] found that microwave blanching was more effective than the conventional method in blunting enzymes (PPO and POD) and retaining nutrients. Recent studies have also reported that microwave energy might break down cell structures, thereby increasing the bioavailability of active natural compounds [15,20].

However, to the best of our knowledge, there are no reports describing the effect of microwave blanching on the drying characteristics and quality attributes of *A. sessiliflorus.* The main objectives of this work are to (i) evaluate the enzyme inactivation efficiency of HMRDB pretreatment; (ii) analyze the effect of HMRDB pretreatment on drying rate, phytochemicals contents, and antioxidant capacity; and (iii) reveal the quality enhancement mechanism of blanching pretreatment. This work aims to provide an innovative approach to increasing the commercial value of *A. sessiliflorus*.

## 2. Materials and Methods

### 2.1. Materials and Reagents

The *A. sessiliflorus* fruits were provided by Dandong Academy of Agricultural Sciences (Liaoning, China). After picking, the fruits were stored in a refrigerator at 4 °C for less than 2 days. Samples of similar size and weight with no mechanical damage were manually selected to ensure uniformity of the experimental results. The initial moisture content of the samples was 82.0 ± 1% on a wet basis (w.b.).

Ethanol for food industry use was obtained from Modern Oriental Technology Development Co., Ltd. (Beijing, China). Methanol, Folin–Ciocalteu, sodium carbonate, gallic acid, rutin, 2,2-azino-bis (3-ethylbenzothiazoline-6-sulfonic acid) diammonium salt (ABTS•), DPPH• free radical, potassium persulfate, guaiacol, catechol, cyanidin-3-glucoside, and sodium hydroxide were purchased from Lanyi Chemical Co., Ltd. (Beijing, China).

### 2.2. HMRDB Pretreatment

The ORW2S-3000R microwave hot-air flow rolling dryer was installed in the College of Engineering of China Agricultural University (Beijing, China). The schematic diagram of the equipment is shown in Figure 1. Based on the pre-experiment, the conditions of microwave hot-air flow rolling dry-blanching treatment were as follows: microwave power of 4 W/g, hot-air speed of 0.5 m/s, hot-air temperature of 65 °C, and rolling bed speed of 5 rpm. A batch (400 g) of the sample was placed in the equipment and then blanched for various durations (2, 4, 6, and 8 min). After blanching, half of the samples were removed and stored in a refrigerator at 4 °C for further analysis, while the other half was further dried as described below.

### 2.3. Drying Kinetics

The drying was performed in the microwave hot-air flow rolling dryer (Figure 1), with a hot-air temperature of 65 °C, microwave power of 0.6 W/g, hot-air wind speed of 0.5 m/s, and rolling bed speed of 5 rpm; samples were dried until the moisture content was about 10.0 ± 0.1% (w.b.). The sample dried without blanching was used as the control group. The moisture ratio (*MR*) is calculated by Equation (1) [5]:

(1) $$MR=\frac{M_{t}}{M_{0}}$$

where *M*_0_ and *M_t_* are the initial moisture content and moisture content at drying time *t* on a dry basis, respectively.

Henderson–Pabis, Logarithmic, Midilli–Kucuk, and Verma models (Table 1) were used to fit the drying curves of *A. sessiliflorus* fruits [21]. The fitting performance of the model was determined by regression coefficient (*R*^2^), root mean square error (*RMSE*), and chi-square (*χ*^2^) [5].

### 2.4. PPO and POD Activity

The polyphenol oxidase (PPO) and peroxidase (POD) extracts were prepared following the method described by Su et al. [18] with some modifications. Briefly, 3 g of fresh or blanched samples was added to 15.0 mL of phosphate buffer solution (0.1 M, pH = 6.5) and ground in an ice bath. The mixture was centrifuged at 6000 rpm for 30 min, and the supernatant was collected for the determination of PPO and POD activity.

For the POD assay, the reaction mixture consisted of 3.0 mL of guaiacol solution (25 mM), 0.2 mL of enzyme extract, and 0.2 mL of H_2_O_2_ (0.5 M). Absorbance values at 470 nm were recorded using a spectrophotometer (TU-1810PC, Puxi Instrument, Beijing, China). For the POD assay, the reaction consisted of 4.0 mL of acetic acid-sodium acetate buffer (50 mM, pH = 5.5), 1.0 mL of catechol solution (50 mM), and 0.1 mL of enzyme extract. Absorbance values were measured at 410 nm. One unit of enzyme activity was defined as a 0.01 unit change in absorbance value per gram of sample per min. The relative enzyme activity was calculated by Equation (2):

(2) $$Relative\;enzyme\;activity\;\left(\%\right)=\frac{A_{t}}{A_{0}}\times 100$$

where *A_t_* and *A*_0_ represent the enzyme activity of treated and fresh samples, respectively.

### 2.5. Determination of Weight Loss

The weight loss was determined by weighing the samples before and after blanching using an electronic balance (model JJ1000, 0.01 precision, G&G Co., Raleigh, NC, USA), according to Equation (3) [13]:

(3) $$Weight\;loss\;\left(\%\right)=\frac{W_{0}-W_{1}}{W_{0}}\times 100$$

where *W*_0_ is the initial weight and *W*_1_ is the weight of the pretreated sample.

### 2.6. Determination of Rehydration Ratio

The dried samples were placed in a beaker containing 100 mL of distilled water with a water bath temperature of 50 °C. After soaking for 2 h, the samples were taken out and then weighed after the surface water was absorbed by filter paper. The rehydration ratio was calculated using Equation (4) [5]:

(4) $$Rehydration\;ratio=\frac{M_{1}}{M_{2}}$$

where *M*_1_ and *M*_2_ are the masses of dried fruits before and after rehydration (g), respectively.

### 2.7. Microstructure and Porosity

The microstructure of the samples was observed by a scanning electron microscope (SU3500, Hitachi Co., Tokyo, Japan) with an acceleration voltage of 15.0 kV. The surface of the sample was coated with gold before observation.

The cross-sectional pictures obtained from the scanning electron microscope were converted to 8-bit gray images and a certain threshold was then set to obtain binary images using ImageJ software (National Institutes of Health, Bethesda, MD, USA). The porosity was calculated by Equation (5) [18]:

(5) $$Porosity=\frac{A_{all}-A_{b}}{A_{all}}\times 100\%$$

where $A_{all}$ is the area of the whole picture and $A_{b}$ is the area of the black part.

### 2.8. Determination of Phytochemical Content and Antioxidant Activity

#### 2.8.1. Sample Extraction

Preparation of extracts to determine the phytochemical content and antioxidant activity of *A. sessiliflorus* was performed according to the method reported by Chen et al. [22] with some modifications. The dried samples were crushed by a high-speed multifunctional crusher (Q-250 A3 type, Bingdu Electric Co., Ltd., Shanghai, China) and passed through an 80-mesh sieve. Sample powder (1.0 g) was dissolved in a methanol aqueous solution (80%) and left in the dark for 16 h. The mixture was then extracted ultrasonically at 40 kHz for 30 min, centrifuged at 7000 rpm for 15 min, and the supernatant was filtered through a 0.45 μm organic filter.

#### 2.8.2. Total Polyphenol Content (TPC)

Total phenolic content (TPC) was determined by the Folin–Ciocalteu method [22]. Briefly, 0.1 mL of extract was added to a 10 mL volumetric flask containing 7.9 mL of distilled water and 0.5 mL of the Folin–Ciocalteu reagent. The mixture was shaken for 5 min before adding 1.5 mL of sodium carbonate solution (20 g/L). The absorbance at 765 nm was read after standing for 2 h at 25 °C on a spectrophotometer (TU-1810PC, Puxi Instrument, China). The results were expressed as mg gallic acid equivalents per gram of sample in terms of dry weight (mg GAE/g DW).

#### 2.8.3. Total Flavonoid Content (TFC)

The TFC was measured according to the method of Xu et al. [23] with some modifications. First, a 2 mL diluted solution of powdered extract was put in a 10 mL volumetric flask. Subsequently, 0.3 mL of NaNO_2_ (5%) was added to the volumetric flask and, after standing for 6 min, 0.3 mL of AlCl_3_·6H_2_O (10%) was added. After soaking for 6 min, 2 mL of NaOH (4%) was added. Next, 5.4 mL of distilled water was added to the reaction flask after 15 min and mixed well. The absorbance of the reaction mixture was read at 510 nm. The results were expressed as rutin equivalents in terms of dry weight (RE mg/g DW).

#### 2.8.4. Total Anthocyanin Content (TAC)

The pH differential absorbance method was applied for the determination of TAC. Briefly, 1 mL of extract was diluted with 9 mL of two different buffer systems: potassium chloride buffer (pH = 1.0, 0.025 M) and sodium acetate buffer (pH = 4.5, 0.4 M). The absorbance values of the dilutions at 510 nm and 700 nm were determined using a spectrophotometer (TU-1810PC, Puxi Instrument, China) against a blank with distilled water. The TAC was calculated using Equation (6) [24]:

(6) $$\mathrm{TAC}=\left[\frac{\left(A\times M_{w}\right)}{\left(\varepsilon \times 1\right)}\right]\times D_{f}\times 1000$$

(7) $$A={\left(A_{510}-A_{700}\right)}_{pH1.0}-{\left(A_{510}-A_{700}\right)}_{pH4.5}$$

where *M_w_* is the relative molecular mass of cyanidin-3-glucoside (449.38 g/mol), ε is the molar absorptivity of cyanidin-3-glucoside (26,900 L/mol. cm), $D_{f}$ is the dilution factor of the extracts, and *A* is the absorbance. The anthocyanin content was expressed as mg cyanidin-3-glucoside equivalent per 100 g of sample in terms of dry weight (Cy-3G/100 g DW).

### 2.9. Determination of Antioxidant Activity

For the DPPH• assay, 2 mL of extract was mixed with 4.0 mL of 0.1 mmol/L DPPH• solution, and the mixture was allowed to stand for 30 min in the dark at room temperature. The absorbance of the reaction mixture was then read at 517 nm against 80% methanol. The DPPH• scavenging activity was calculated by using Equation (8) [25]:

(8) $$S=\lfloor 1-\frac{A_{i}-A_{j}}{A_{0}}\rfloor \times 100$$

where *S* is the DPPH• radical scavenging ability (%), $A_{i}$ is the absorbance of the DPPH• and sample extract solution, $A_{j}$ is the absorbance of the sample, and $A_{0}$ is the absorbance of the DPPH• methanol solution.

For the ABTS• assay, 100 μL of extract was reacted with the ABTS• radical working solution (3.9 mL) in the dark (25 °C) for 6 min. The absorbance of the reaction mixture was then read at 734 nm against 80% methanol. The ABTS• scavenging activity of the samples was calculated by Equation (8), and the results were expressed as Trolox equivalent antioxidant capacity in terms of dry weight (μmol TE/g DW) [26].

### 2.10. Data Analysis

The significance of the differences was analyzed using Origin 2021 and SPSS 17.0 software by one-way ANOVA at a significant level of 95% (*p* < 0.05). All experiments were conducted in triplicate and the results were expressed as means ± standard deviation.

## 3. Results and Discussion

### 3.1. Effect of HMRDB on PPO and POD Enzyme Inactivation

**Figure 2:**
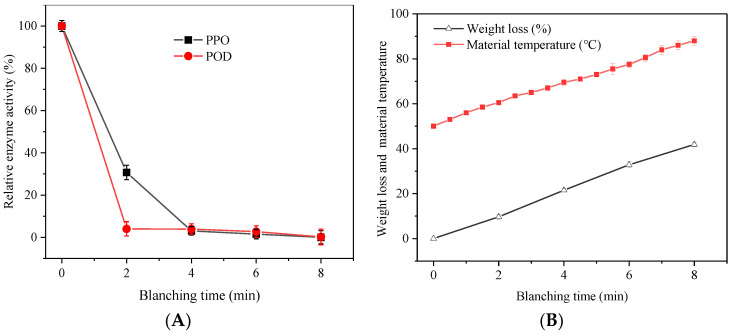
Changes in enzyme activity (**A**) and changes in weight loss and material temperature (**B**) in *A. sessiliflorus* fruits blanched for different durations. PPO, polyphenol oxidase; POD, peroxidase.

The inactivation degree of polyphenol oxidase (PPO) and peroxidase (POD) can be used as a benchmarking characteristic for the adequacy of the blanching treatment [8]. This is because PPO and POD were proven to be responsible for tissue browning during fruit and vegetable processing [27]. They may also affect other quality attributes, such as antioxidant properties and bioactive compound content [15]. The evolution of the relative activity of POD and PPO in *A. sessiliflorus* fruits with different HMRDB pretreatment times is shown in Figure 2A. The POD and PPO activity of fresh samples were 9.86 and 0.89 Abs∙min^−1^g^−1^, respectively, and the activity of POD was significantly (*p* < 0.05) higher than that of PPO. PPO and POD activity decreased gradually with the extension of blanching time. When blanched for 2 min, the relative activity of PPO and POD decreased significantly (*p* < 0.05), from 100% to 30.71% and 4%, respectively. After blanching for 8 min, PPO and POD were almost completely inactivated. The inactivation of PPO and POD was positively correlated with the temperature of the samples because high temperatures disrupted the protein structure of the enzymes [9]. The surface temperature of the material was 60.5, 69.5, 77.5, and 88.00 °C after blanching for 2, 4, 6, and 8 min (Figure 2B). Zhu and Pan [28] showed that most of the PPO was destroyed when the temperature was between 60 and 80 °C, and the inactivation temperature of POD was relatively higher. Similar results were also reported by Su et al. [18], who found that the PPO and POD enzymes were completely inactivated after blanching for 9 min. Okonkwo et al. [13] reported that the POD inactivation time of microwave blanching (5 and 7 min) was shorter than that of hot water (7 and 9 min) and infrared blanching (18 and 21 min). Therefore, HMRDB pretreatment is an acceptable technique for efficient inactivation of POD and PPO enzymes in food.

### 3.2. Effect of HMRDB on Weight Loss

The effects of different HMRDB pretreatment times on the weight loss of *A. sessiliflorus* fruits are shown in Figure 2B. It can be seen that weight loss gradually increased with the increase in blanching time. The weight loss of the samples after pretreatment for 2, 4, 6, and 8 min was 9.64, 21.50, 32.84, and 41.85%, respectively. This was likely due to water evaporation coinciding with increased blanching temperature; the temperature of the material was 88.0 °C when blanched for 8 min. Okonkwo et al. [13] found similar weight loss behavior during the microwave blanching of Irish potatoes. Ruiz-Ojeda and Penas [29] reported that the weight loss of microwave-blanched green beans was higher than that of hot water-blanched beans. They also found that weight loss increased with increases in microwave power and treatment time. The higher weight loss helped to shorten drying time, which was related to the change in drying time for samples after blanching.

### 3.3. Effect of HMRDB on Drying Kinetics

**Figure 3:**
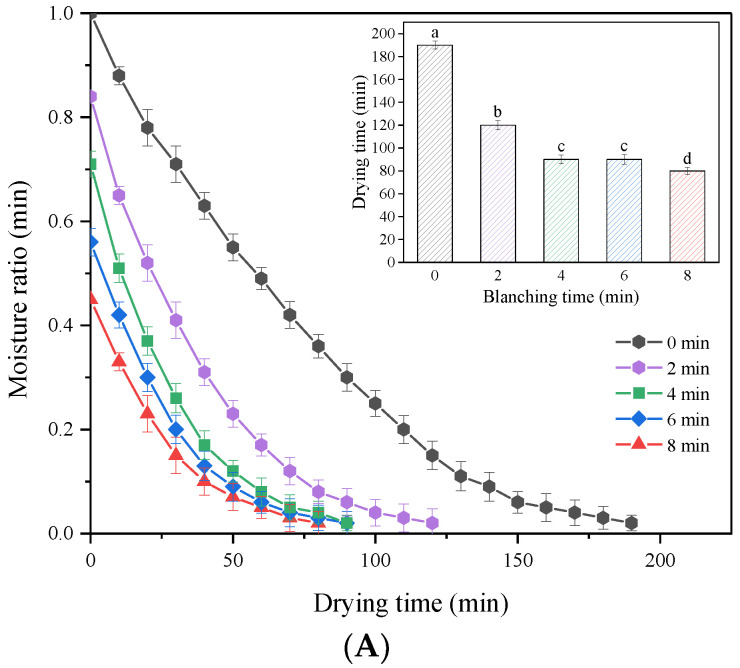

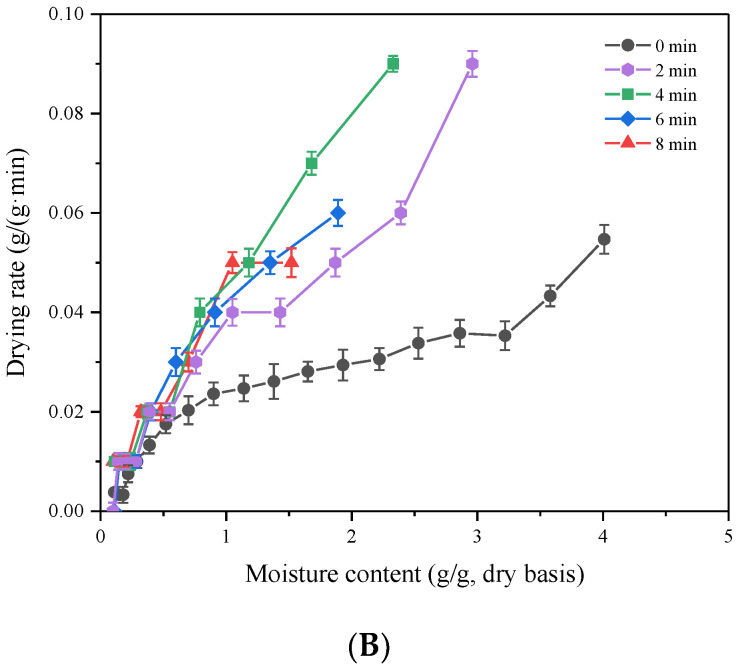
Moisture ratio (**A**) and drying rate curves (**B**) of *A. sessiliflorus* fruits with different blanching durations. Different letters indicate significant differences (*p* < 0.05) according to the Duncan test.

The effects of different HMRDB pretreatment times on the drying kinetics of *A. sessiliflorus* fruits are shown in Figure 3. Due to evaporation, the drying process resulted in a progressive drop in the moisture ratio (Figure 3A). Compared to untreated samples (190 min), blanching for 2 to 8 min reduced drying time by 36.80 to 57.89%. The drying time reduced as the blanching time increased. The decrease in drying time may be attributable to the evaporation of free water, the increase in porosity, and the breakdown of the cell wall [10]. Blanching as a pretreatment removed a substantial amount of free water prior to drying, hence shortening the drying time using microwave hot-air rolling. These results were similar to the results of Su et al. [18], who found that that the drying time of *Pleurotus eryngii* decreased from 195 min to 95 min when HMRDB was applied.

Figure 3B depicts the effects of various blanching times on the drying rate of the samples. It can be observed that the drying rate of the blanched samples first increased and then decreased as their moisture content decreased. The drying rate of the unblanched samples was significantly lower than that of the blanched samples (*p* < 0.05), with prolonged drying time for the unblanched samples. Interestingly, increasing the blanching time (over 6 min) did not lead to a higher drying rate, which might be caused by the damage in plant cell walls due to extended microwave heating. Su et al. [18] reported that the blanching treatment resulted in the formation of porous structures in tissue cells during the destruction of macromolecules such as pectin and proteins, which facilitated the migration of water during the drying process. The surface layer that covers the surface of *A sessiliflorus* is a crucial element in limiting water diffusion [30]. Blanching damaged the structure of these surface layers, allowing for rapid evaporation of the fruit’s moisture, which enhanced the sample’s drying rate. Wang et al. [30] observed that cracks formed in pepper skin after hot blanching and that the dense waxy structure of the skin was destroyed. Jiang et al. [17] found that hot water blanching and microwave blanching of *Agaricus bisporus* reduced drying time by 25% and 41%, respectively. Therefore, microwave blanching allows for higher drying rates than conventional techniques, which may mean lower processing costs.

**Figure 4:**
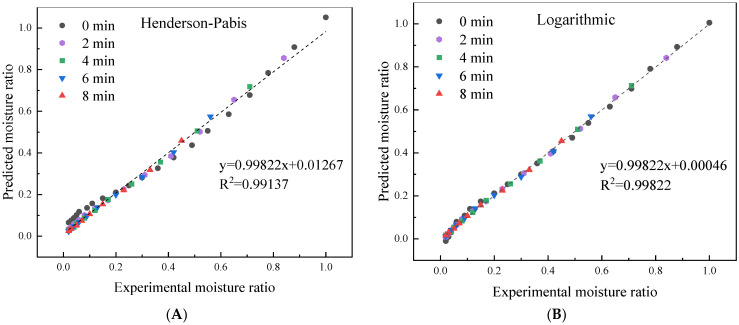

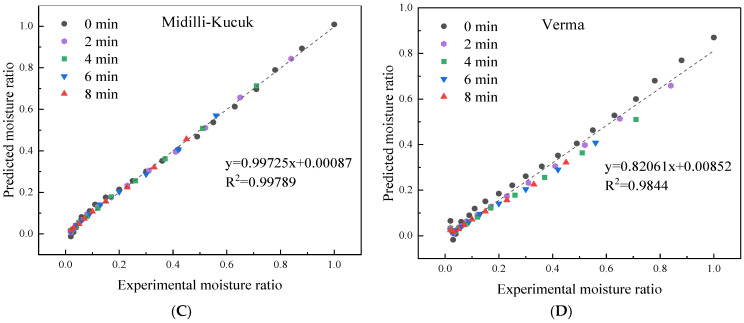
Experimental and predicted moisture ratio of *A. sessiliflorus* fruits with different blanching durations. (**A**) Henderson–Pabis model; (**B**) Logarithmic model; (**C**) Midilli–Kucuk model; and (**D**) Verma model.

The constants and performance of different drying models for the fitted curves are shown in Table 1. Compared to Henderson–Pabis, Midilli–Kucuk, and Verma, the Logarithmic model had the best fitting performance, with high *R*^2^ (0.9972–0.9995), low χ^2^ (3.66 × 10^−5^–2.99 × 10^−4^), and low *RMSE* (0.0061–0.0173) values. The Logarithmic model also showed good fitting for drying of barbunya peach [31] and green bell pepper [32]. Predicted and experimental moisture ratio (*MR*) plots for the drying model are shown in Figure 4. The slope and intercept of the regression curve are closer to 1 and 0, respectively, meaning better prediction accuracy. This further verified the fitness of the Logarithmic model, with a slope of 0.99822 and an intercept of 0.00046.

### 3.4. Effect of HMRDB on Microstructure

**Figure 5:**
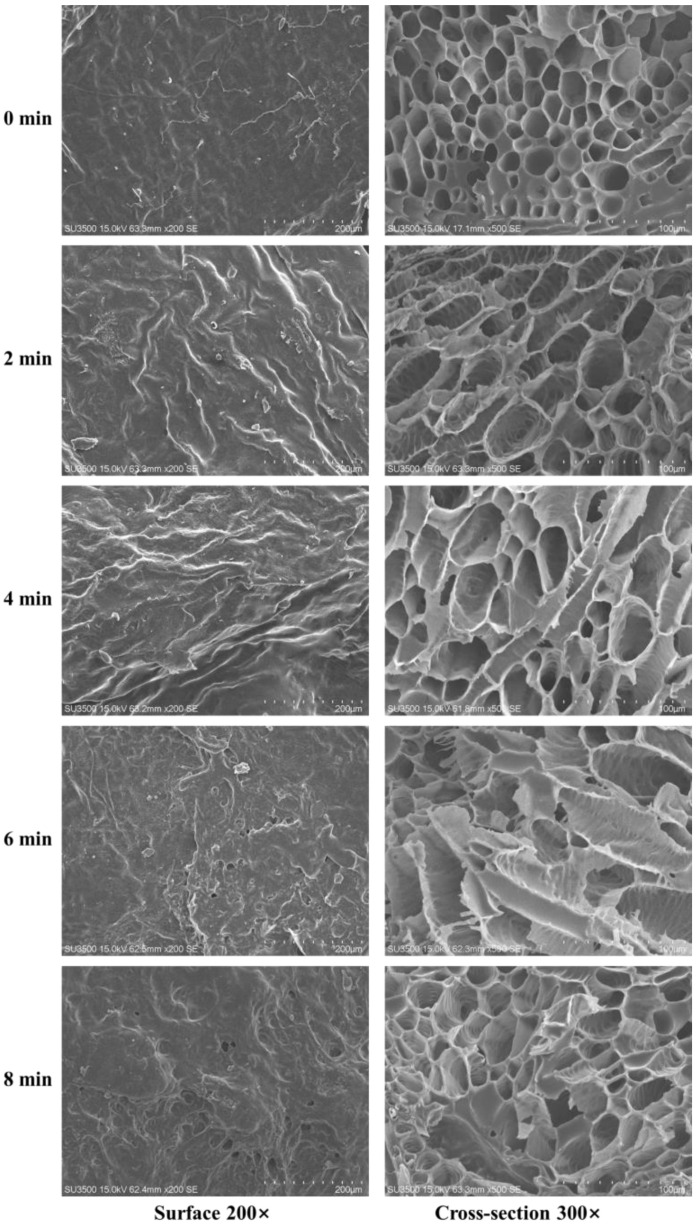
Scanning electron microscope images of *A. sessiliflorus* fruits with different blanching treatments. The magnification level is ×200 for the surface and ×300 for the cross-section.

The microstructure of the dried sample’s surface and cross-section was observed using SEM, as shown in Figure 5. The surface of the unblanched samples (×200 magnification) was comparatively smooth and flat, whereas the surface of the blanched samples was heavily wrinkled and rough. In the later stages of blanching (6 and 8 min), pores were found, which enabled the migration of moisture throughout the drying process. The shrinkage of the samples was attributed to the loss of water and the destruction of the cell structure [5]. The waxy layer structure on the surface was the primary impediment to the diffusion of moisture during drying and blanching caused the degradation of the waxy layer structures, hence boosting the drying rate. Similar results were found by Wang et al. [30], who observed cracks on the surface of peppers following blanching, with the number of cracks increasing as the blanching duration increased.

**Figure 6:**
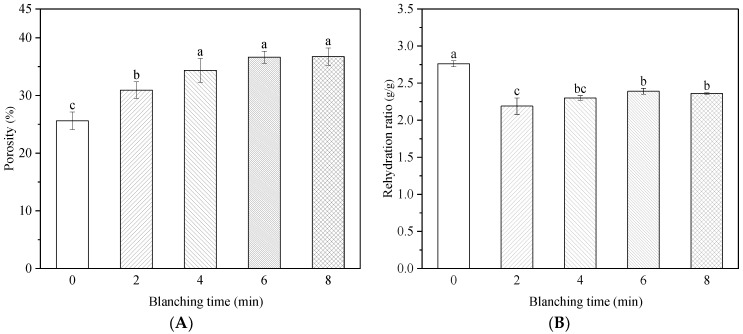
Porosity (**A**) and rehydration properties (**B**) of *A. sessiliflorus* fruits with different blanching durations. Different letters indicate significant differences (*p* < 0.05) according to the Duncan test.

The cross-sectional scans of the samples (×300 magnification) revealed that the tissue of the unblanched samples remained intact, with distinct cell outlines and no severe collapse; however, the cell structure of the blanched samples was severely disturbed. In general, the cell wall consists of matrix polysaccharides (such as cellulose) and structural polysaccharides (such as pectin and hemicellulose), which form a meshwork structure that promotes the maintenance of the essential cell shape [33]. It was reported that the tissue hardness of carrots decreased after blanching in hot water at 95 °C for 2 min. The molecular length and height of water-soluble, chelating, and alkali-soluble pectin decreased, the long chain of chelating pectin disappeared, and the network structure of alkali-soluble pectin was destroyed [34]. Ni et al. [35] showed that blanching caused the degradation and dissolution of tissue cell wall pectin, resulting in decreased cell turgor and intercellular adhesion. In addition, due to the rapid evaporation of water, the internal pressure of the cells increases during the microwave blanching process, resulting in the rupture of the cell walls [17]. The porosity of the samples increased from 25.60% to 36.73% when blanching time was increased from 0 to 10 min (Figure 6A). The amount of water vapor inside the sample increased during the microwave treatment, which resulted in an increase in the internal pressure in the cells and promoted the formation of pores [5]. Porous structures form an essential moisture dispersion channel, that influences drying rate and physicochemical properties. However, prolonged blanching could lead to the collapse of cell structures and reduction in porosity [9,18].

### 3.5. Effect of HMRDB on Rehydration Ratio

The rehydration property of dried *A. sessiliflorus* is also important because the product is often rehydrated before further processing [5]. As shown in Figure 6, the rehydration ratio of the blanched samples was significantly (*p* < 0.05) lower than that of the unblanched sample. Blanching disrupted the integrity of the cells, which limited the water-holding capacity of the sample during rehydration [36]. Ojediran et al. [37] reported that cracks in the waxy layer of Hog plum after hot water blanching and excessive shrinkage during drying of the sample hindered its water-holding capacity, thus reducing its rehydration ratio. The rehydration ratio gradually increased with the increase in blanching time, which was related to the increased amount of porous structures inside the material (which will be discussed in detail in the next section) [5].

### 3.6. Effect of HMRDB on Phytochemical Content

**Figure 7:**
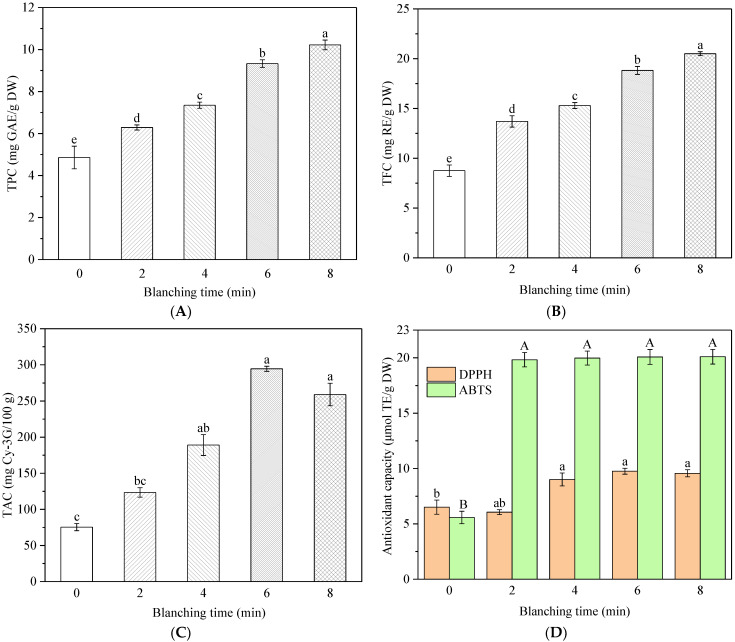
Changes in the total polyphenol content (TPC) (**A**), total flavonoid content (TFC) (**B**), total anthocyanin content (TAC) (**C**), and antioxidant capacity (**D**) of *A. sessiliflorus* fruits with different blanching treatments. Different letters indicate significant differences (*p* < 0.05) according to the Duncan test.

**Figure 8:**
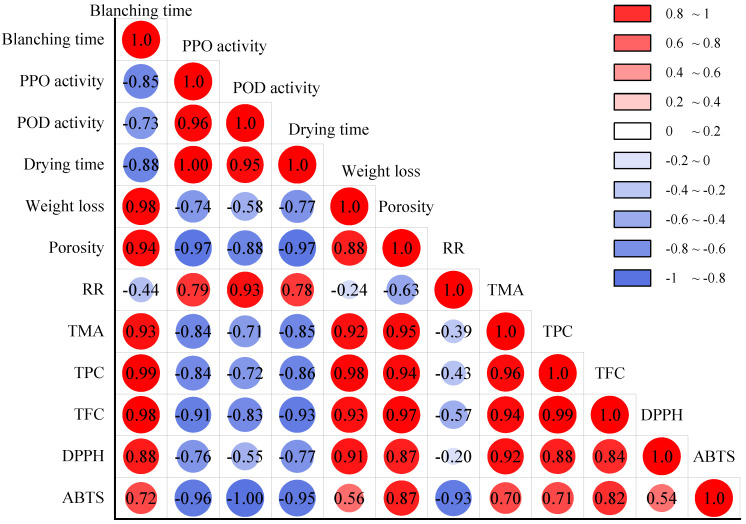
Pearson correlation analysis on different parameters of *A. sessiliflorus* fruits with different blanching treatments. PPO, polyphenol oxidase; POD, peroxidase; RR, rehydration ratio; TPC, total polyphenol content; TFC, total flavonoid content; TAC, total anthocyanin content.

Phenolic substance compounds, including flavonoids, are widely present in plant cells in the free or bound form [15]. The changes in TPC and TFC for *A. sessiliflorus* fruits after HMRDB pretreatment and drying are shown in Figure 7. It was obvious that the blanched samples had significantly (*p* < 0.05) higher TPC and TFC in comparison to the unblanched samples, as shown in Figure 7A,B. Similar results were also found in apricots [9], *pleurotus eryngii* [18], and carrots [10]. Phenolics are highly unsaturated, and they are easily oxidized and degraded during the drying process [38]. The drying time was negatively correlated with TPC (*r* = −0.86) and TFC (*r* = −0.93) (Figure 8). By shortening the drying time, blanching may have reduced the probability of phenolic chemicals undergoing oxidative degradation. Heras-Ramirez et al. [39] reported that the inactivation of polyphenol oxidases enhanced the stability of phenolic compounds since these enzymes may be involved in the enzymatic oxidative degradation of phenolic compounds.

Correlation analysis showed that TPC and TFC were negatively correlated with PPO (*r* = −0.84, −0.91) and POD (*r* = −0.72, −0.83) (Figure 8). In addition, the TPC and TFC steadily increased as the time of HMRDB pretreatment increased. HMRDB pretreatment destroys the cellular structure, consequently releasing a large number of phenolic chemicals from the cells and dissolving them in the extractant [15].

Anthocyanins are water-soluble natural pigments with an unstable structure that is easily affected by environmental temperature, pH, oxidants, enzymes, metal ions, light, and other factors [24]. Total anthocyanin content (TAC) in the samples after blanching and drying treatment is shown in Figure 7C. TAC increased and then decreased with increasing blanching treatment time, and the lowest TAC (75.43 mg Cy-3G/100 g DW) was found in the unblanched sample, which was attributed to the oxidative degradation of total anthocyanin during the prolonged drying time. The highest content was obtained at 6 min of blanching (294.44 mg Cy-3G/100 g DW), which was about 3.9 times higher than that of the unblanched sample. However, TAC of the sample blanched for 8 min decreased by 12% compared to the 6 min sample. Mendez-Lagunas et al. [40] reported that anthocyanins are degraded by contact with enzymes due to structural changes in the material (slumping and porosity), thus enzyme inactivation might explain the higher retention of anthocyanins in blanched samples compared to untreated samples. The blanching treatment increased the extraction rate of anthocyanins, probably due to microwave penetration of the cell structure, which caused the outflow of anthocyanins from the cells, thus increasing the content [15]. However, prolonged pretreatment softened the tissue structure of *A. sessiliflorus* fruits and severely damaged the cell wall, leading to the oxidative decomposition of anthocyanins in the drying process.

### 3.7. Effect of HMRDB on Antioxidant Activity

The changes in antioxidant activity in *A. sessiliflorus* fruits after pretreatment and drying were measured using the DPPH• and ABTS• methods, as shown in Figure 7D. The scavenging ability of DPPH• and ABTS• free radicals gradually increased with pretreatment time, except for the samples that were blanched for 2 min. The highest DPPH• and ABTS• radical scavenging activity was observed in the samples blanched for 8 min, with values of 9.57 and 20.09 μmol TE/g DW, respectively. This suggested that HMRDB pretreatment increased the antioxidant activity of the samples, which was similar to the results of previous studies. Mendez-Lagunas et al. [40] found that the antioxidant activity of pepper increased from 29 to 42 μmol TE/g after microwave blanching, which may be due to the conversion of phenolics during the drying process producing other derivatives with higher antioxidant activity. Deng et al. [9] reported that blanching increased the antioxidant capacity of apricots because the intracellular proteins and cell matrix released antioxidants more efficiently. In addition, DPPH• and ABTS• were positively correlated with TPC, TFC, and TAC values, as shown in Figure 8. This suggested that the antioxidant properties may be related to specific bioactive compounds, such as phenolic acid which acts as a free radical scavenger in the oxidation process. Similar results were reported in the study of apricots [9] and sliced persimmon [25]. Overall, the dried samples after blanching had higher antioxidant capacity than the unblanched samples. The overall quality of microwave-bleached products is acceptable. Therefore, HMRDB could be a suitable technique to preserve the phytochemical content and antioxidant capacity of *A. sessiliflorus* fruits.

## 4. Conclusions

Microwave hot-air flow rolling dry-blanching (HMRDB) was applied to the pretreatment of *A. sessiliflorus* fruits before drying. The results showed that the polyphenol oxidase and peroxidase of the samples were almost completely inactivated after blanching for 8 min. Blanching pretreatment reduced the drying time of the samples by 36.8 to 57.89%. The Logarithmic model fitted the drying curves better (*R*^2^ = 0.9972−0.9995) than the Henderson–Pabis, Midilli–Kucuk, and Verma models. The cellular structure of the blanched samples was disrupted by significant puckering and increased porosity. Moreover, the blanched samples had higher levels of total phenols, total flavonoids, total anthocyanins, and antioxidant activity than the unblanched control sample. The retention of phytochemical content was related to enzyme inactivation and shortened drying time. The current study demonstrates that HMRDB technology has the potential to improve the drying quality and drying efficiency of *A. sessiliflorus.*

## Figures and Tables

**Table 1:** Parameters and fitting performance of different drying models.

Blanching Time (min)		0	2	4	6	8
Henderson–Pabis y = *a* exp (−*k t*)	*k*	0.0146	0.0267	0.0352	0.0354	0.0366
	*a*	1.0512	0.8557	0.7187	0.5748	0.4589
	*R* ^2^	0.9825	0.9961	0.9985	0.9964	0.9977
	*χ* ^2^	1.79 × 10^−3^	2.98 × 10^−4^	9.26 × 10^−5^	1.38 × 10^−4^	5.93 × 10^−5^
	*RMSE*	0.0423	0.0173	0.0096	0.0118	0.0077
Logarithmic MR = *a* exp (−*k t*) + *c*	*k*	0.0098	0.0232	0.0324	0.0324	0.0337
	*a*	1.2002	0.8882	0.7350	0.5894	0.4703
	*c*	−0.1949	−0.0465	−0.0223	−0.0196	−0.0149
	*R* ^2^	0.9972	0.9991	0.9995	0.9977	0.9986
	*χ* ^2^	2.99 × 10^−4^	7.90 × 10^−5^	3.66 × 10^−5^	1.02 × 10^−4^	4.23 × 10^−5^
	*RMSE*	0.0173	0.0089	0.0061	0.0101	0.0065
Midilli–Kucuk MR = *a* exp (−*k t*) + *b t*	*k*	0.0114	0.0245	0.0335	0.0337	0.0349
	*a*	1.0085	0.8434	0.7135	0.5706	0.4559
	*b*	−0.0007	−0.0003	−0.0002	−0.0002	−0.0001
	*R* ^2^	0.9967	0.9988	0.9994	0.9975	0.9985
	*χ* ^2^	3.59 × 10^−4^	9.76 × 10^−5^	4.27 × 10^−5^	1.12 × 10^−4^	4.5 × 10^−5^
	*RMSE*	0.0190	0.0099	0.0065	0.0106	0.0068
Verma y = exp(−*k t*) + (1−*a*) exp (−*g t*)	*k*	0.0117	0.0214	0.0285	0.0271	0.0277
	*a*	1.0180	1.1596	1.2884	1.4314	1.5453
	*g*	−0.0103	0.0074	0.0178	0.0195	0.0223
	*R* ^2^	0.9933	0.9991	0.9995	0.9976	0.9984
	*χ* ^2^	6.55 × 10^−4^	6.41 × 10^−5^	2.82 × 10^−5^	8.33 × 10^−5^	3.58 × 10^−5^
	*RMSE*	0.0256	0.0080	0.0053	0.0091	0.0060

## Data Availability

Data are contained within the article.
